# Crystalline Lens Thickness Changes in Myopia Children During Long‐Term Orthokeratology Treatment

**DOI:** 10.1155/joph/1623610

**Published:** 2026-02-16

**Authors:** Bohua Jiang, Yifei Meng, Zuocheng Wang, Shuaixi Pan, Pengfei Wang, Sufang Qie, Xiaohui Tong, Zhipeng yan

**Affiliations:** ^1^ The Department of Ophthalmology, The Third Hospital of Hebei Medical University, 139 Ziqiang Road, Shijiazhuang, China, hebmu.edu.cn; ^2^ School of Management, Shijiazhuang Tiedao University, Shijiazhuang, 050043, China, stdu.edu.cn; ^3^ School of Medical Imaging, Hebei Medical University, 48 Donggang Road, Shijiazhuang, China, hebmu.edu.cn

**Keywords:** axial length elongation, crystalline lens thickness, myopia, orthokeratology

## Abstract

**Objectives:**

To investigate ocular parameters associated with axial length (AL) growth in children wearing orthokeratology lens during 2 years of follow‐up.

**Methods:**

This is a retrospective study. Medical records of 46 patients who underwent orthokeratology treatment for 2 years were reviewed. Baseline variables included age at initiation of orthokeratology wear, spherical equivalent (SE), central corneal thickness (CCT), and the flat and steep keratometry of corneal principal meridians. The changes in anterior chamber depth (ACD) and crystalline lens thickness (CLT) were also analyzed. The contributions of all variables to the AL elongation were assessed using univariate and multivariate regression analyses.

**Result:**

CLT and AL significantly increased after 2 years of orthokeratology wear compared with baseline (both *p* < 0.01), whereas ACD did not significantly change compared with baseline (*p* = 0.301). Univariate analyses showed that a reduced rate of AL elongation was found in children who were older age (*p* = 0.02), had greater SE (*p* = 0.026), thicker CCT at baseline (*p* = 0.027), and more increase in CLT (*p* = 0.019) in 2 years. Furthermore, greater SE at baseline and more increase in CLT were associated with less elongation of AL during 2 years of follow‐up in multivariable analyses (*p* = 0.044 and 0.034).

**Conclusion:**

CLT was significantly increased in the first year and keep stable in the second year in children with orthokeratology wearing. Greater baseline SE and more increase in CLT were associated with less elongation in AL during orthokeratology wear.

## 1. Background

Over the past decade, myopia has emerged as a highly prevalent eye condition worldwide. The increasing prevalence of childhood myopia is now recognized as a major public health concern. Myopia has reached epidemic proportions in East and Southeast Asia, with prevalence rates among young adults reaching 80%–90% [1]. Effectively inhibiting or even slowing the progression of myopia remains a challenge. Various interventions have been evaluated for this purpose in children, including bifocal/multifocal spectacle lenses, orthokeratology (Ortho‐K) contact lenses, and pharmacological treatments [2]. The efficacy of these myopia control options varies. A global survey of myopia management attitudes and strategies among eye‐care practitioners reported that Ortho‐K was perceived as one of the most effective methods for myopia control [3].

Ortho‐K lenses work by inducing central corneal flattening and mid‐peripheral corneal steepening that provides clear foveal vision while simultaneously causing a myopic shift in peripheral retinal defocus. This peripheral myopic retinal defocus caused by Ortho‐K is hypothesized to be responsible for reductions in myopia progression in children [4]. Several studies indicate that key anterior corneal surface parameters including flat‐k, steep‐k, and K‐max demonstrate significant reductions with Ortho‐K wear both in the short‐ and long‐term periods [5–7]. Except for anterior corneal surface alterations, a few of studies have examined changes in the ocular anterior segment during Ortho‐K wear, particularly over the short term. Chen and Shen demonstrated that 6 months of Ortho‐K wear led to a reduction in anterior chamber depth (ACD) and an increase in crystalline lens thickness (CLT) [8]. In a 12‐month prospective study, Qin Zhu et al. reported that Ortho‐K lenses increased CLT while demonstrating no significant effect on ACD [9]. Tang et al. further concluded that a shortening of axial length (AL) by 0.1 mm was accompanied by a thickening of the CLT and a reduction in ACD within 1 year of Ortho‐K treatment [10]. They proposed that an increase in CLT could serve as a clinical indicator for superior myopia control efficacy. This view finds support in our earlier 12‐month study, where a significant CLT thickening was also observed in children undergoing Ortho‐K and was associated with a slower increase in AL. In contrast, ACD showed no significant change during the follow‐up period [6]. Therefore, Ortho‐K appears to exert effects beyond mere corneal reshaping, influencing both corneal tissue and anterior segment structure within a short‐term period.

Ortho‐K for myopia control typically requires long‐term treatment in children, often lasting for many years. However, the long‐term trend of CLT, whether it continues to increase, stabilizes, or recovers toward baseline, remains unclear. Moreover, evidence linking long‐term CLT changes to axial elongation is scant. To address this, the present study extends our prior work by observing CLT changes over a 2‐year Ortho‐K treatment period and analyzing their relationship with AL elongation. These findings may deepen the understanding of Ortho‐K’s influence on the anterior segment and its underlying control mechanisms.

## 2. Methods

This study followed the Declaration of Helsinki and was approved by the ethics committee of the Hebei Medical University. A retrospective chart review was performed by analyzing the medical records of all participants who were initially fitted with overnight Ortho‐K lenses in the Department of Ophthalmology at the Third Hospital of Hebei Medical University between June 2021 and January 2022 and finished their 2 follow‐ups between June 2023 and January 2024. Consent was obtained from both the patient and their parents to use their medical records for study purposes.

All patients included in this study underwent routine examination in every 3 months on time during 2 years of follow‐up. The following inclusion criteria were required for a patient to be included in this retrospective study: 1) patient aged between 8 and 14 years at the start of Ortho‐K lenses’ use; 2) initial myopic refractive error between −0.75D and‐5.00D and with‐the‐rule astigmatism (≤ 1.00 D). All patients underwent routine eye examinations and refractive status assessment before wearing the Ortho‐K lenses. They were fitted with either spherical or toric designed 4‐zone Ortho‐K lenses (alpha, Japan). The refractive error of all patients was fully corrected such that the monocular best corrected visual acuity was better than 6/7.5 on a Snellen eye chart which was achieved after treatment stabilization. All measurements, medical record documentation, and patient interactions were performed by one ophthalmologist from the initiation of the Ortho‐K lenses and during every follow‐up, thereafter including the 24‐month follow‐up. Only the right eye data were used for statistical analysis.

The baseline data collected for analysis included the age at initiation of Ortho‐K wear, gender, SE, central corneal thickness (CCT), and the flat and steep keratometry (flat‐K and steep‐K) of the corneal principal meridians. The changes in ACD, CLT, and AL between baseline and follow‐up periods were recorded. CCT, and flat‐ and steep‐K of the corneal principal meridians were examined using a Pentacam analysis system (Oculus, Wetzlar, Germany) at baseline and follow‐up periods. ACD (the distance between the front surface of the cornea and the front surface of the lens), CLT, and AL were examined using a commercially available optical biometry (OA‐2000, Tomey, Nagoya, Japan) at baseline and every follow‐up periods. Five consecutive measurements were collected from each subject for each measure, and the values were averaged.

## 3. Statistical Analysis

Data were analyzed using SPSS 22.0 (SPSS Inc., Chicago, IL, USA) and Python 3.7.4. The Kolmogorov–Smirnov (KS) test was used to determine the distribution of the data, which included flat‐K, steep‐K, CCT, ACD, CLT, and AL values at baseline and at 12‐month and 24‐month follow‐up (*p* > 0.05). Paired samples’ *t*‐test and Wilcoxon signed rank test were used to compare variables for normal and non‐normal distributed variables, respectively. To control the risk of multiple comparisons, the significance level was adjusted to 0.0167 through the Bonferroni correction. The primary outcome measure of this study was the change in AL among baseline. Analyzed variables associated with AL changes included the age at initiation of Ortho‐K wear, gender, SE, CCT, flat‐K, steep‐K, and changes in ACD and CLT. Factors with a statistical significance level of *P* < 0.05 were selected to input into the multivariate regression analysis model. The strength of association for significant variables was represented using beta values, 95% confidence intervals, adjusted *R* ^2^ values, and *p* values.

## 4. Result

A total of 46 right eyes from 46 subjects (32 girls and 14 boys) were included in this study. The average age at the initiation of Ortho‐K lens wear was 10.07 ± 1.65 years. The average baseline SE was −2.25 (‐3.75, −1.50) D. The average AL was 24.67 ± 0.76 mm at baseline. Detailed subject characteristics are provided in Table 1.

**TABLE 1:** Patients and study eye characteristics (*n* = 46 patients).

Demographic characteristics (*n* = 46 eyes)	Mean ± SD or No.(%)	Range
Age (years)	10.07 ± 1.65	8 to 15

*Gender*		
Female	32	69.57%
Male	14	30.43%
SE (DS)	−2.25(‐3.75, −1.50)	−0.75 to −5.50
Flat‐K	42.41 ± 1.14	40.30 to 45.70
Steep‐K	43.82 ± 1.15	41.30 to 47.10
ACD (mm)	3.76 ± 0.19	3.30 to 4.10
CCT (μm)	549.74 ± 27.97	490 to 612
CLT (mm)	3.39 ± 0.15	3.10 to 3.62
AL (mm)	24.67 ± 0.76	22.40 to 26.29

*Note:* DS = diopters of sphere, K=keratometry, and ACD = anterior segment depth.

Abbreviations: AL = axial length, CCT = central corneal thickness, CLT = crystalline lens thickness, and SE = spherical equivalent.

### 4.1. Anterior Segment Changes After 2 Years of Ortho‐K Treatment

First, The KS test was used to analyze the distribution of flat‐K, steep‐K, CCT, ACD, CLT, and AL values at baseline and at 12‐month and 24 month follow‐up. All data recorded were normally or non‐normally distributed, as shown in sTable 1. As expected, the flat‐K, steep‐K, and CCT were significantly decreased at 1‐year and 2‐year follow‐up compared with the baseline during Ortho‐K treatment. Although total ACD was not significantly changed after 2 years compared with the baseline (*p* = 0.301), it was significantly decreased by 0.025 ± 0.067 mm in the first year (*p* = 0.013) and subsequently significantly increased by 0.015 ± 0.040 mm in the second year (*p* = 0.015). In terms of CLT, it was significantly increased by 0.027 ± 0.050 mm in the first year compared with the baseline (*p* < 0.01). Subsequently, it increased slightly but not significantly in the second year (*p* = 0.191). The AL was significantly increased by 0.272 ± 0.205 mm in the first year compared with baseline (*p* < 0.01). Subsequently, it continued to significantly increase by 0.236 ± 0.146 mm in the second year compared with that in the first year of follow‐up (*p* < 0.01) (Table 2).

**TABLE 2:** Comparison analysis for ocular biometric parameters at baseline and at 1 year and 2 years following OK lens wear.

Variable	Baseline	One year	Two years	*p* ^@^	Effect size^@^	*p* ^#^	Effect size^#^	*p* (%)	Effect size (%)
ACD (mm)	3.760 ± 0.193	3.735 ± 0.204	3.750 ± 0.200	0.013^∗^	0.380	0.301	0.154	0.015^∗^	0.374
CLT (mm)	3.392 ± 0.150	3.420 ± 0.170	3.426 ± 0.170	< 0.01^∗^	0.549	< 0.01^∗^	0.677	0.191	0.196
AL (mm)	24.665 ± 0.761	24.937 ± 0.733	25.173 ± 0.713	< 0.01^∗^	1.322	< 0.01^∗^	0.870	< 0.01^∗^	0.871
Flat‐K (D)	42.411 ± 1.140	40.558 ± 1.427	40.397 ± 1.632	< 0.01^∗^	1.424	< 0.01^∗^	1.359	0.052	0.289
Steep‐K (D)	43.824 ± 1.153	41.802 ± 1.575	41.963 ± 1.871	< 0.01^∗^	1.466	< 0.01^∗^	1.082	0.811	0.036
CCT (μm)	549.74 ± 27.969	526.70 ± 26.705	528.61 ± 26.600	< 0.01^∗^	3.123	< 0.01^∗^	2.330	0.112	0.301

*Note:* Mean values are presented in mean ± standard deviation (SD). Effect sizes are calculated using Cohen’s D or coefficient of rank correlation. % Between postoperative 1 year and postoperative 2 years.

Abbreviations: ACD = anterior segment depth, AL = axial length, CLT = crystalline lens thickness.

^∗^ *p* < 0.0167: statistically significant.

^@^Between baseline and respective postoperative 1 years.

^#^Between baseline and respective postoperative 2 years.

### 4.2. Variables Associated With AL Elongation During 2 Years of Ortho‐K Treatment

In this study, variables associated with AL elongation included age at initiation of Ortho‐K wear, gender, baseline SE, CCT, flat‐K and steep‐K principal meridians, and changes in CLT and ACD were analyzed. At 1‐year follow‐up, univariate analysis showed age at initiation of Ortho‐K wear, baseline SE, CCT, the changes in ACD and CLT were significantly associated with AL elongation. However, gender, flat‐K, and steep‐K principal meridians did not show obvious association. Further multivariate analysis showed that the decreased ACD and greater SE are associated with the smaller AL elongation (sTable 2 and sTable 3). At 2‐year follow‐up, univariate analysis showed age at initiation of Ortho‐K wear, baseline SE, CCT, and changes in CLT were significantly associated with AL elongation. But gender, flat‐K, and steep‐K principal meridians did not displaye significant association with AL elongation (Table 3 and Figure 1).

**TABLE 3:** Univariate regression analyses of different independent variables on axial length elongation during 2 years of follow‐up.

Variable	Value (mean)	*B* value	*R* ^2^	Adjusted *R* ^2^	*p* value	95% confidence interval	
Age (years)	10.07 ± 1.65	−0.064	0.117	0.097	0.020^∗^	−0.117	−0.010
SE (DS)	−2.625 ± 1.35	0.075	0.108	0.088	0.026^∗^	0.010	0.141
Flat‐K(D)	42.411 ± 1.14	−0.018	0.005	−0.018	0.651	−0.100	0.063
Steep‐K(D)	43.824 ± 1.15	−0.007	0.001	−0.022	0.859	−0.088	0.074
CCT (μm)	549.74 ± 27.97	−0.004	0.106	0.086	0.027^∗^	−0.007	0.000
Change of CLT (μm)	0.0346 ± 0.051	−2.071	0.118	0.098	0.019^∗^	−3.792	−0.350
Gender	F:32 M:14	−0.057	0.008	−0.015	0.566	−0.258	0.143

*Note:* SE = spherical equivalent, DS = diopters of sphere, K = keratometry, CCT = central corneal thickness, and CLT = crystalline lens thickness.

^∗^ *p* < 0.05 = statistically significant.

FIGURE 1Scatterplots showing correlations of axial length change at 24 months of follow‐up, with the age at initiation of Ortho‐K wear (a), spherical equivalent (SE) (b), central cornea thickness (CCT) (c), and the change of crystalline lens thickness (CLT) (d) after univariate regression analysis.

**Figure:**
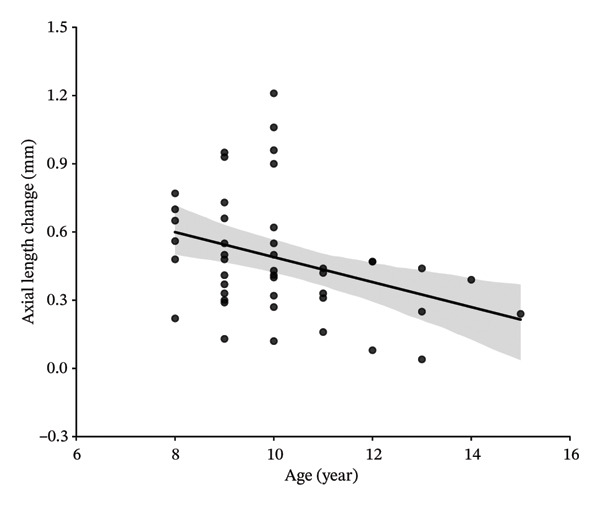
(a)

**Figure:**
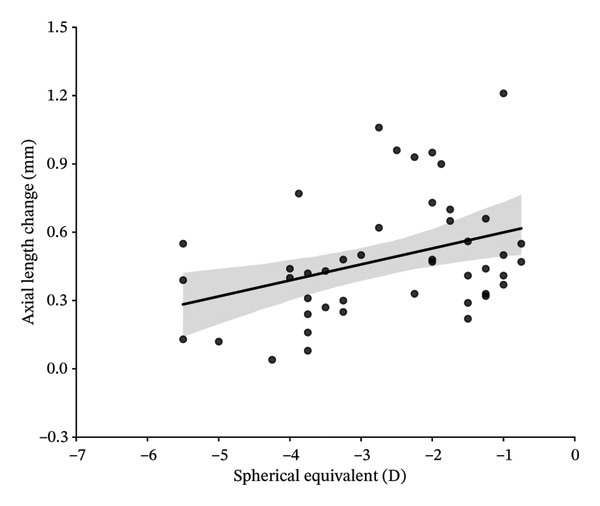
(b)

**Figure:**
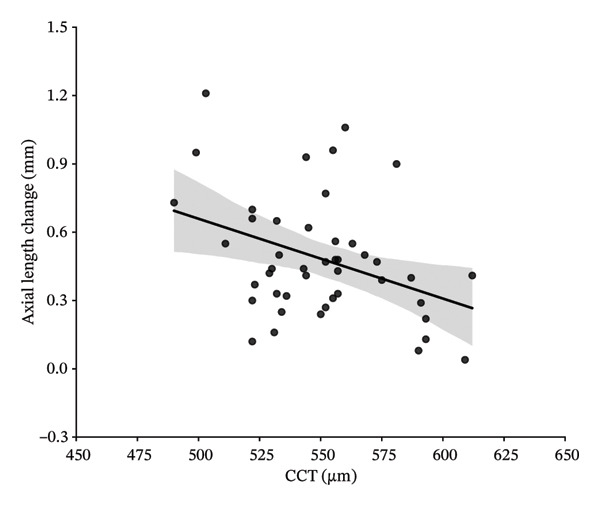
(c)

**Figure:**
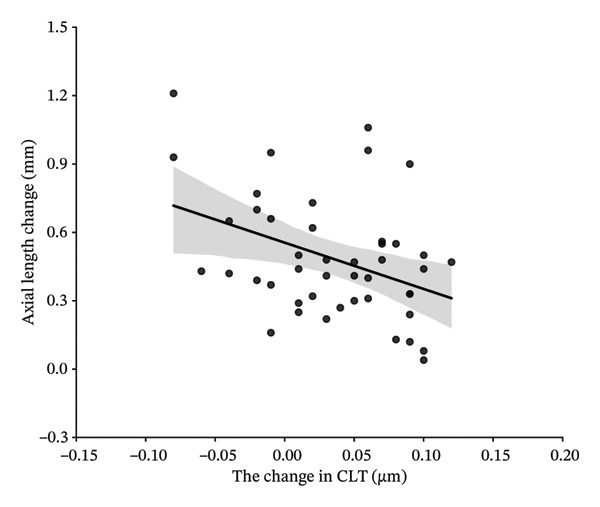
(d)

Then, four variables including age at initiation of Ortho‐K wear, baseline SE, CCT, and changes in CLT were further analyzed by a multivariate analysis to determine their association with AL growth in the 2‐year follow‐up. As shown in Table 4, greater baseline SE and increased CLT were significantly associated with less increase in AL (*P* = 0.044 and 0.034). Age at initiation of Ortho‐K wear and CCT did not show a statistically significant association with AL growth (*p* = 0.193 and 0.319) (Table 4).

**TABLE 4:** Multivariable regression analysis showing the strength of association between the independent variables and axial length growth during 2 years of follow‐up.

Variable	*B* value	*p* value	95% confidence interval	
Change of CLT (μm)	−0.303	0.034^∗^	−3.505	−0.149
SE(DS)	0.286	0.044^∗^	0.002	0.129
Age(years)	−0.198	0.193	−0.093	0.019
CCT (μm)	−0.156	0.319	−0.005	0.002
Final model	*R* ^2^ = 0.198	Adjusted *R* ^2^ = 0.161		

*Note:* DS = diopters of sphere.

Abbreviations: CCT = central corneal thickness, CLT = crystalline lens thickness, SE = spherical equivalent.

^∗^ *p* < 0.05 = statistically significant.

## 5. Discussion

Ortho‐K is considered as one of the most effective clinical options for myopia control in the young population. This conclusion is supported by multiple studies reporting significantly slowed myopia progression in school‐aged children undergoing Ortho‐K treatment [11]. The prevailing hypothesis for Ortho‐K’s efficacy is the “peripheral refraction theory.” Ortho‐K induces a flattened central cornea and steepened midperipheral cornea and then leading to a circular area of increased peripheral myopia on the corneal surface [11].

Multiple randomized and nonrandomized controlled trials have shown a 32%–43% reduction in axial elongation in children undergoing Ortho‐K treatment relative to those wearing regular spectacles [11–13]. In cases of high myopia, Ortho‐K treatment has been shown to achieve up to a 63% reduction in the rate of axial elongation [14]. Determining the optimal candidates for Ortho‐K remains a challenge in clinical practice, as treatment efficacy varies markedly between individuals. While some patients achieve excellent myopia control, others exhibit little to no response despite attaining good daytime uncorrected distance vision acuity. Many studies have demonstrated several factors, including baseline age of Ortho‐K wear, baseline spherical equivalent, rate of myopia progression before initiating Ortho‐K wear, pupil size, peripheral refractive profile, retinal topography (shape), and lens design, may contribute to this difference [6].

In a 2‐year randomized clinical trial, Cho et al. reported that older children undergoing Ortho‐K treatment exhibited slower axial elongation than younger children [12]. Further supporting this, Santodomingo‐Rubido et al. reported that older age was significantly associated with less axial elongation over a 2‐year period in Ortho‐K wearers based on their univariate and multivariate analyses [15]. In the current study, patients with older baseline age also associated with the less AL elongation in the 2‐year follow‐up.

Furthermore, the current study revealed that greater baseline SE was associated with slower axial elongation at 2‐year follow‐up during Ortho‐K wear. This finding aligns with most studies showing that Ortho‐K is more effective at slowing axial growth in children with higher baseline myopia [16–18]. The possible reason is a greater degree of corneal steepening in the midperiphery of higher myopic eyes during Ortho‐K wear, which leads to a greater myopic peripheral defocus before retina and thus further slowing the AL elongation.

The anterior segments, including ACD and CLT during Ortho‐K treatment, were observed by a few studies recently. Chen and Shen observed the anterior segment changes in 6 months during Ortho‐K treatment and found that ACD significantly decreased in the Ortho‐K group compared with those in the control group; CLT in the Ortho‐K group were thicker than those in the control group [8]. In a 12‐month study, Zhouyue Li et al. further reported significant alterations in anterior segment parameters, with a decrease in ACD and an increase in CLT from baseline [19]. In line with the aforementioned studies, the current data also show a decrease in ACD and an increase in CLT at the 12‐month follow‐up compared to baseline. Recently, Tang et al. reported that thickened CLT and decreased ACD were observed in AL reduction eyes during Ortho‐K treatment in 12 months but do not significantly change in groups of AL elongation ≥ 0.2 mm per year [10]. They further concluded that CLT and ACD were not associated with AL elongation by univariate and multivariate analyses in both groups. While most studies confirm alterations in ACD and CLT during Ortho‐K, a few have reported no significant change in these parameters in either short‐ or long‐term investigations [20, 21].

Our previous study showed that the CLT was significantly increased and associated with smaller AL elongation in 1‐year follow‐up [6]. The current study validated this phenomenon and further investigated the association between CLT changes and AL elongation in 2 years during Ortho‐K treatment for the first time. Notably, CLT thickening was attenuated in the second year and did not reach statistical significance. Critically, however, the total CLT increase over the 2 years remained a significant multivariate correlate of slower axial growth.

The underlying mechanism driving CLT changes during Ortho‐K treatment remains unclear. Shih et al. revealed that the crystalline lens became thinner between the ages of 7 and 11, subsequent increases in the lens thickness with age, and myopic eye growth induces the lens to compensate by becoming much thinner than normal eye growth [22]. As a result, Ortho‐K treatment may effectively antagonize this trend of thinning during myopic eye growth.

Similar phenomena have been found in the choroid thickness changes during Ortho‐K treatment. In a one‐year study, Li et al. reported an increase in choroidal thickness that correlated with axial elongation [19]. In their 2 years of randomized clinic trial, Ortho‐K significantly improved the choroidal thickness till 18 months of the follow‐up period. However, this effect diminished at the 2 year follow‐up period [23]. In a longitudinal study, Wen et al. reported that the efficacy of myopia control exhibited a progressive decline over a 24‐month period during Ortho‐K wear [24]. Hiraoka et al. further demonstrated that Ortho‐K provided no further reduction in myopia progression beyond the initial 3‐year treatment period [16]. It is plausible that the temporal attenuation of Ortho‐K efficacy is associated with concurrent choroidal thinning.

In the present study, the myopia control effect of Ortho‐K showed no evidence of attenuation over 2 years, as evidenced by comparable axial elongation rates between the first and second years (0.23 mm; 0.27 mm, *p* = 0.19). This sustained efficacy may be related to the maintenance of CLT. To the best of our knowledge, this represents the longest investigation (2 years of follow‐up) to date analyzing the association between CLT changes and AL elongation. While our previous 1‐year study established that increased CLT was associated with slower AL growth, this extended analysis confirms that greater CLT remains a significant correlation of reduced AL elongation over 2 years even though CLT keep stable without further increase in the second year. Intriguingly, a previous study demonstrated that lens thickness decreased significantly (by 0.02 mm) one month after discontinuing Ortho‐K treatment compared with measurements taken at the 12‐month visit [19]. As a result, Ortho‐K treatment should be responsible for the maintenance of lens thickening. Although it is difficult to explain the direct connection between lens thickness changes and Ortho‐K wear, the initial increase in CLT during the first year may represent a compensatory response to changes in anterior segment geometry induced by Ortho‐K, such as corneal flattening and altered peripheral refraction. After 1 year, the eye reaches a new balance, where further lenticular thickening is no longer necessary to maintain optical and structural balance. This stabilization could reflect an adaptive endpoint in the eye’s response to consistent mechanical and optical stimuli from overnight Ortho‐K wear.

The potential physiological mechanism of crystalline lens changes during Ortho‐K wear is still unclear. However, it is conceivable that crystalline lens could not be indefinitely thicker during Ortho‐K treatment. Takahiro Hiraoka reported that a significant difference in the annual increase in AL was observed until the third year between the OK and control groups, but not in the fourth and fifth years [16]. Whether CLT could recover to baseline levels, similar to choroidal thickness fluctuation in a longer follow‐up period, is one of the important issues for understanding ocular biometric changes during Ortho‐K treatment.

This study has several limitations. Its retrospective design lacked a control group wearing single‐vision spectacles for direct comparison. The follow‐up period was relatively short, and the sample size was modest. Furthermore, potential variability in Ortho‐K lens fit and differences in patient compliance over the treatment period could influence outcomes. Future prospective studies with larger cohorts, longer follow‐up, randomized controlled designs, and standardized lens fitting protocols are needed to clarify the long‐term ocular biometric changes under Ortho‐K and identify factors influencing its sustained efficacy.

## 6. Conclusions

This study provides compelling evidence that CLT was significantly increased in the first year and keep stable in the second year in children with Ortho‐K wear. Greater baseline SE and more increase in CLT were associated with less elongation of AL during Ortho‐K wear in 2 years of the follow‐up period.

## Author Contributions

Bohua Jiang and Yifei Meng contributed equally to this work.

## Funding

The study was supported by the Natural Science Foundation of Hebei Province, H2022206225.

## Disclosure

Crystalline lens thickness changes in myopia children during orthokeratology treatment of the long‐term study.

## Conflicts of Interest

The authors declare no conflicts of interest.

## Supporting Information

Additional supporting information can be found online in the Supporting Information section.

## Supporting information


**Supporting Information 1** sTable 1: Kolmogorov–Smirnov test for ocular biometric parameters.

**Supporting Information 2** sTable 2: Univariate regression analyses of different independent variables on axial length elongation during one‐year follow‐up.

**Supporting Information 3** sTable 3: Multivariable regression analysis showing the strength of association between the independent variables and axial length growth during one‐year follow‐up.

## Data Availability

All the supporting data are included within the article.
