# Supplementary material for: Crystalline Lens Thickness Changes in Myopia Children During Long‐Term Orthokeratology Treatment
Source: J Ophthalmol. 2026 Feb 16;2026:1623610. doi: 10.1155/joph/1623610 (PMC12909614; doi:10.1155/joph/1623610)
Supplement: Supplementary file 1 — Supporting Information 1 sTable 1: Kolmogorov–Smirnov test for ocular biometric parameters. [file JOPH-2026-1623610-s001.docx]

sTable.1 Kolmogorov-Smirnov Test for ocular biometric parameters

| Variable | N | Test Statistic | Asymp. Sig. (2-tailed) |
| --- | --- | --- | --- |
| SE | 45 | 0.135 | 0.035 |
| ACD(0-1) | 45 | 0.077 | 0.200* |
| ACD(0-2) | 45 | 0.105 | 0.200* |
| ACD(1-2) | 45 | 0.093 | 0.200* |
| CLT(0-1) | 45 | 0.107 | 0.200* |
| CLT(0-2) | 45 | 0.104 | 0.200* |
| CLT((1-2) | 45 | 0.121 | 0.090 |
| AL(0-1) | 45 | 0.078 | 0.200* |
| AL(0-2) | 45 | 0.162 | 0.004 |
| AL(1-2) | 45 | 0.172 | 0.002 |
| Flat K(0-1) | 45 | 0.077 | 0.200* |
| Flat K(0-2) | 45 | 0.093 | 0.200* |
| Flat K(1-2) | 45 | 0.224 | ＜0.001 |
| Steep K(0-1) | 45 | 0.094 | 0.200* |
| Steep K(0-2) | 45 | 0.121 | 0.091 |
| Steep K(1-2) | 45 | 0.225 | ＜0.001 |
| CCT(0-1) | 45 | 0.095 | 0.200* |
| CCT(0-2) | 45 | 0.089 | 0.200* |
| CCT(1-2) | 45 | 0.210 | ＜0.001 |

ACD= anterior segment depth, CLT=Crystalline lens thickness, AL=Axial length, Flat K=flat keratometry of the corneal principal meridian, Steep K=steep keratometry of the corneal principal meridian, CCT= central corneal thickness, 0-1=the difference between the 1-year follow-up and baseline, 0-2= the difference between the 2-year follow-up and baseline, 1-2=the difference between the 2-year follow-up and 1-year follow-up.

*. This is a lower bound of the true significance.
