# Supplementary material for: Crystalline Lens Thickness Changes in Myopia Children During Long‐Term Orthokeratology Treatment
Source: J Ophthalmol. 2026 Feb 16;2026:1623610. doi: 10.1155/joph/1623610 (PMC12909614; doi:10.1155/joph/1623610)
Supplement: Supplementary file 2 — Supporting Information 2 sTable 2: Univariate regression analyses of different independent variables on axial length elongation during one‐year follow‐up. [file JOPH-2026-1623610-s003.docx]

sTable 2. Univariate regression analyses of different independent variables on axial length elongation during one year follow up.

| Variable | Value (Mean) | B value | R^2^ | Adjusted R^2^ | P value | 95% Confidence interval | |
| --- | --- | --- | --- | --- | --- | --- | --- |
| Age（years） | 10.07±1.65 | -0.042 | 0.114 | 0.094 | 0.022* | -0.078 | -0.006 |
| SE (DS) | -2.625±1.35 | 0.047 | 0.094 | 0.073 | 0.038* | 0.003 | 0.091 |
| Flat K | 42.411±1.14 | 0.002 | 0.000 | -0.023 | 0.939 | -0.053 | 0.057 |
| Steep K | 43.824±1.15 | 0.010 | 0.054 | 0.003 | 0.721 | -0.044 | 0.064 |
| CCT | 549.74±27.97 | -0.002 | 0.115 | 0.095 | 0.021* | -0.005 | 0.000 |
| Change of ACD (µm) | -0.0254±0.067 | 1.497 | 0.238 | 0.220 | 0.001* | 0.683 | 2.311 |
| Change of CLT (µm) | 0.0276±0.050 | -1.661 | 0.165 | 0.146 | 0.005* | -2.794 | -0.527 |
| Gender | F:32 M:14 | -0.045 | 0.011 | -0.012 | -0.497 | -0.179 | 0.088 |

SE=Spherical equivalent, DS=Diopters of Sphere, K=Keratometry, CCT=Central corneal thickness, ACD=Anterior segment depth, CLT=Crystalline lens thickness.

* p<0.05 = Statistically significant.
