# Supplementary material for: Crystalline Lens Thickness Changes in Myopia Children During Long‐Term Orthokeratology Treatment
Source: J Ophthalmol. 2026 Feb 16;2026:1623610. doi: 10.1155/joph/1623610 (PMC12909614; doi:10.1155/joph/1623610)
Supplement: Supplementary file 3 — Supporting Information 3 sTable 3: Multivariable regression analysis showing the strength of association between the independent variables and axial length growth during one‐year follow‐up. [file JOPH-2026-1623610-s002.docx]

sTable 3. Multivariable regression analysis showing the strength of association between the independent variables and axial length growth during one year follow up.

| Variable | B value | P value | 95% Confidence interval | |
| --- | --- | --- | --- | --- |
| Change of ACD(µm) | 1.440 | 0.001* | 0.655 | 2.224 |
| Change of CLT(µm) | -0.069 | 0.943 | -1.989 | 1.851 |
| SE（DS） | 0.042 | 0.036* | 0.003 | 0.081 |
| Age(years) | -0.001 | 0.551 | -0.049 | 0.027 |
| CCT | -0.001 | 0.329 | -0.003 | 0.001 |
| Final model | R^2^=0.313 | Adjusted R^2^=0.281 | | |

SE=Spherical equivalent, ACD=Anterior segment depth ,CLT=Crystalline lens thickness, DS=Diopters of Sphere, CCT=central corneal thickness.

* p<0.05 = Statistically significant.
